# Metal Complexation of Bis-Chalcone Derivatives Enhances Their Efficacy against Fusarium Wilt Disease, Caused by *Fusarium equiseti*, via Induction of Antioxidant Defense Machinery

**DOI:** 10.3390/plants11182418

**Published:** 2022-09-16

**Authors:** Asmaa El-Nagar, Abdelnaser A. Elzaawely, Tran Dang Xuan, Mohamed Gaber, Nadia El-Wakeil, Yusif El-Sayed, Yasser Nehela

**Affiliations:** 1 Department of Agricultural Botany, Faculty of Agriculture, Tanta University, Tanta 31527, Egypt; 2 Transdisciplinary Science and Engineering Program, Graduate School of Advanced Science and Engineering, Hiroshima University, Hiroshima 739-8529, Japan; 3 Chemistry Department, Faculty of Science, Tanta University, Tanta 31527, Egypt; 4 Center for the Planetary Health and Innovation Science (PHIS), The IDEC Institute, Hiroshima University, Higashi-Hiroshima 739-8529, Japan

**Keywords:** chalcone, metal ion, wilt, soil-borne pathogen, enzymatic antioxidant machinery, non-enzymatic antioxidant machinery

## Abstract

Sweet pepper (*Capsicum annuum* L.) is one of the most widely produced vegetable plants in the world. Fusarium wilt of pepper is one of the most dangerous soil-borne fungal diseases worldwide. Herein, we investigated the antifungal activities and the potential application of two chalcone derivatives against the phytopathogenic fungus, *Fusarium equiseti*, the causal agent of Fusarium wilt disease in vitro and in vivo. The tested compounds included 3-(4-dimethyl amino-phenyl)-1-{6-[3-(4 dimethyl amino-phenyl)-a cryloyl]-pyridin-2-yl}-propanone (DMAPAPP) and its metal complex with ruthenium III (Ru-DMAPAPP). Both compounds had potent fungistatic activity against *F. equiseti* and considerably decreased disease progression. The tested compounds enhanced the vegetative growth of pepper plants, indicating there was no phytotoxicity on pepper plants in greenhouse conditions. DMAPAPP and Ru-DMAPAPP also activated antioxidant defense mechanisms that are enzymatic, including peroxidase, polyphenole oxidase, and catalase, and non-enzymatic, such as total soluble phenolics and total soluble flavonoids. DMAPAPP and Ru-DMAPAPP also promoted the overexpression of *CaCu-SOD* and *CaAPX* genes. However, *CaGR* and *CaMDHAR* were downregulated. These results demonstrate how DMAPAPP and Ru-DMAPAPP could be employed as a long-term alternative control approach for Fusarium wilt disease as well as the physiological and biochemical mechanisms that protect plants.

## 1. Introduction

The genus *Capsicum* sp. (*Family: Solanaceae*) is native to the Americas but is cultivated worldwide for its chili pepper and bell pepper fruits. *Capsicum* consists of more than fifty thousand cultivars grown worldwide that belong to 20–27 species [1], with five major species which are widely cultivated, including *C. annuum*, *C. baccatum*, *C. chinense*, *C. frutescens*, and *C. pubescens* [2]. Among these five major species, sweet pepper (*Capsicum* *annuum* L.), also known as bell pepper, is the most widely cultivated species. In 2020, approximately 36 million tons of sweet pepper were produced globally, including production by China (16.7 million tons) with 46% of the total, followed by Mexico (2.8 million tons) and Indonesia (2.8 million tons) [3]. In the same year, Egypt grew 58,402 hectares of sweet pepper, yielding 18.0748 tons per hectare with a total production of 1.1 million tons, putting Egypt in sixth place among the top sweet pepper producers after Turkey (2.6 million tons) and Spain (1.5 million tons) [3].

Many diseases and disorders can interfere with pepper production. For instance, sweet pepper is susceptible to numerous economically important diseases [4]. These diseases include several fungal, bacterial, viral, and nematode diseases that can infect all parts of the pepper plant, including the foliage, stems, roots, fruit, and young seedlings [5]. Among these diseases, root rots and wilt diseases are considered the most serious problems in most pepper-producing areas [5] worldwide. Several phytopathogenic fungi and fungi-like organisms may be involved in causing root rots and wilt diseases in pepper, such as Phytophthora root rot (caused by the fungus-like organism *Phytophthora capsica*), Verticillium wilt (caused by *Verticillium dahliae* and *V. albo-atrum*), Rhizoctonia root rot (caused by *Rhizoctonia solani*), and Fusarium wilt (caused by *Fusarium* sp.) [6].

Fusarium wilt of pepper is one of the most destructive diseases that reduces crop yields [7] and causes economic yield losses of up to 70% [8,9,10]. Fusarium wilt of sweet pepper was reported in the USA, Argentina, China, Italy, and many other countries. This disease was proposed to be associated with the causal organism *Fusarium oxysporum* [5], particularly *F. oxysporum* f. sp. *capsici*. However, it was also reported to be associated with some other Fusarium species [11,12], such as *F. moniliforme*, *F. palidoroseum* [13], and *F. solani* [14]. Recently, *F. equiseti* was also reported to be associated with chili wilt from Kashmir (Northern Himalayas) [15].

Generally, the management of Fusarium wilt of pepper starts before crop planting to improve plant health and control the yield losses due to disease [5]. Various management strategies have been developed for the control of Fusarium wilt disease. These strategies include, but are not limited to, (i) physical control such as soil solarization, heating-based soil disinfection, biofumigation, and biological soil disinfection; (ii) cultural control, such as growing pathogen-free seeds and resistance cultivars, crop rotation, soil amendment, the rouging and eradication of diseased plants and alternative hosts, and grafting; and (iii) biological control using different bioagents [5]. However, chemical control using agrochemicals such as fumigants and fungicides is the most commonly used strategy to control this disease. Despite the effectiveness of these compounds in eliminating the pathogen and increasing crop quality, the usage of agrochemicals has been seriously questioned due to their negative impact on the environment, human and animal health, and other non-targeted microorganisms [16]. Moreover, the extensive utilization of fungicides leads to the development of resistant populations of the pathogen.

Unfortunately, none of these strategies has been able to entirely suppress the disease due to the broad host range and the sustained survival of the pathogen in the soil, even though there are no resistant/tolerant sweet pepper cultivars. Therefore, sustainable management strategies for the Fusarium wilt disease of sweet pepper and other Solanaceae crops require searching for effective alternatives to conventional fungicides that are also more environmentally friendly [17]. These alternative strategies include the synthesis and implementation of sustainable ecofriendly natural compounds, such as chalcone derivatives, to reduce the usage of fungicides entirely or at least partially.

Chemically, chalcones are *1,3–diphenyl-2-propene-1-one* molecules containing dual aromatic rings that are linked to each other via a carbon bridge system and polyhydroxylated in the aryl rings [18,19]. Chalcones can be found naturally in foods, fruits, vegetables, tea, and other plants and have been proven to have significant biological activities, such as antibacterial, antioxidant, antiviral, and antifungal properties [20,21,22,23]. For instance, chalcone derivatives inhibited the mycelial growth of several phytopathogenic fungi, such as *Helminthosporium maydis*, *Sclerotinia sclerotiorum*, *Rhizoctonia solani*, *Gibberella zeae*, and *Botrytis cinerea* [24], as well as the fungi-like organism *Phytophthora infestans* [25]. Recently, twenty piperazine fragment-containing chalcone derivatives showed strong antifungal activities against the soil-borne phytopathogen *Rhizoctonia solani* [26]. However, the physiological, biochemical, and molecular mechanisms behind the antifungal role of chalcones as well as their physiological effects on treated plants are poorly understood.

More than ten years ago, we synthesized the 3-(4-dimethylamino-phenyl)-1-{6-[3-(4-dimethylamino-phenyl)-acryloyl]-pyridin-2-yl}-propanone (DMAPAPP; a bis-chalcone derivative) [27], and recently we designed its metal complex with manganese (Mn-DMAPAPP), iron (Fe-DMAPAPP), and ruthenium (Ru-DMAPAPP) using DMAPAPP as a ligand [28]. Moreover, we studied the structural elucidation, spectral behavior, thermal properties, and catalytic activity of both compounds. However, their antimicrobial activity and their potential biological roles are insufficiently studied.

In the current study, we evaluated DMAPAPP and its metal complex Ru-DMAPAPP against *F. equiseti*, the causal agent of Fusarium wilt disease in sweet pepper. We presume that the metal complexation of bis-chalcone (DMAPAPP) might enhance its efficacy against *F. equiseti* in a yet unidentified mechanism. Moreover, our preliminary studies showed that DMAPAPP and Ru-DMAPAPP have a strong antioxidant capacity that was expressed by higher scavenging activity (%) in vitro. We hypothesize that the antioxidant activity of DMAPAPP and Ru-DMAPAPP might influence the antioxidant defense machinery of *F. equiseti*-infected pepper plants. We believe that bis-chalcone derivatives might be a novel ecofriendly therapeutic strategy to battle Fusarium wilt disease, not only in sweet pepper but also in other solanaceous crops.

## 2. Results

### 2.1. Metal Complexation of DMAPAPP Boosts Its Antioxidant Activity

**Figure 1:**
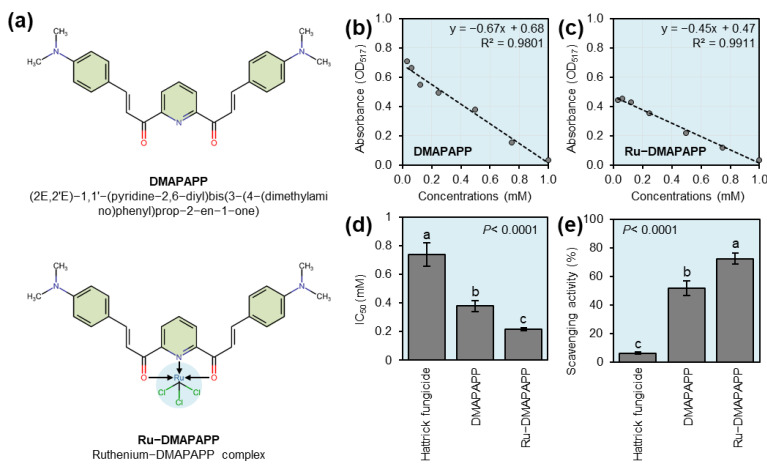
In vitro antioxidant activities of two bis-chalcone derivatives, DMAPAPP and its metal complex with ruthenium (Ru-DMAPAPP). (**a**) Chemical structures of DMAPAPP and Ru-DMAPAPP. (**b**,**c**) Standard curves of in vitro antioxidant activities using different concentrations (0.03, 0.06, 0.13, 0.25, 0.50, 0.75, and 1.00 mM) of DMAPAPP and Ru-DMAPAPP, respectively. (**d**) IC_50_ and (**e**) scavenging activity (%) of DMAPAPP and Ru-DMAPAPP, respectively. Values represent the means ± standard deviations (means ± SD). Different letters indicate statistically significant differences between treatments using the Tukey–Kramer honestly significant difference test (Tukey HSD; *p* < 0.05).

The potential antioxidant activities of two bis-chalcone derivatives, DMAPAPP (Figure 1b) and its metal complex with ruthenium (Ru-DMAPAPP; Figure 1c) were examined in vitro. Briefly, DMAPAPP and Ru-DMAPAPP showed higher antioxidant capacity (as expressed by a lower IC_50_ value) than the control and fungicide (Figure 1d). In other words, the scavenging activity (%) of DMAPAPP and Ru-DMAPAPP were significantly increased (51.91 ± 5.19 and 72.62 ± 3.63%, respectively; Figure 1e), whereas the positive control “Hattrick” showed very low scavenging activity (6.23 ± 0.68%).

### 2.2. Pathogenicity Test of Fusarium Isolates on Pepper Plants

**Figure 2:**
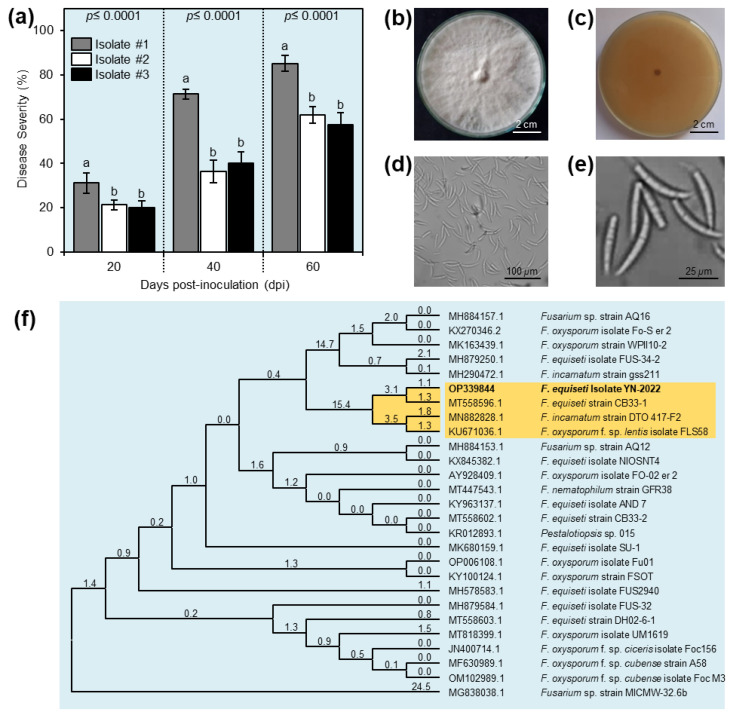
Pathogenicity, morphological characterization, and molecular identification of *Fusarium equiseti* isolates. (**a**) Disease severity (%) of different *F. equiseti* isolates on pepper seedlings (cultivar Top star) under greenhouse conditions. Values represent the means ± standard deviations (means ± SD). Different letters indicate statistically significant differences between treatments using the Tukey–Kramer honestly significant difference test (Tukey HSD; *p* <0.05). (**b**,**c**) Appearance of the fungal colony on potato dextrose agar (PDA) media from the top and the bottom of the petri dish, respectively, after 7 days of incubation at 27 ± 2 °C. (**d**,**e**) Microscopic typical appearance of hyaline, cylindrical, and multiple-cell macroconidia of *F. equiseti*. (**f**) Maximum-likelihood phylogenetic tree using ITS-5.8S rDNA sequence of *F. equiseti* isolate YN-2022 (GenBank accession No. OP339844) (highlighted in bold along with its sequence from 25 reference strains/isolates retrieved from the recent available data in the National Center for Biotechnology Information (NCBI) GenBank (https://www.ncbi.nlm.nih.gov/; accessed on 2 September 2022).

The three isolates demonstrated various degrees of ability to elicit plant wilt under greenhouse conditions. Isolate #1 had the most severe disease severity (31.18 ± 4.51, 71.31 ±2.17, and 85.14 ± 3.54%) at 20, 40, and 60 days post-inoculation (dpi). Meanwhile, at 20, 40, and 60 dpi, isolates #2 and #3 caused the least disease severity, with no significant differences between them (Figure 2a). According to these findings, it was obvious that isolate #1 was the most pathogenic isolate; therefore, it was chosen for all subsequent experiments.

### 2.3. Morphological Characterization

Isolate #1 showed typical morphological and microscopic characteristics with the phytopathogenic fungus *Fusarium*. The fungal colony on PDA media was milky white (Figure 2b) and reverse beige or light brown (Figure 2c). The fungal colony took 7 days to fully cover the surface of a 9 cm Petri dish when grown on PDA at room temperature (27 ± 2 °C). The aerial mycelium cultures were moderately puffy to fluffy (Figure 2b). Microscopic examination revealed hyaline and septate hyphae, hyaline macroconidia (Figure 2d,e) that were cylindrical with a slight bend at the tip and 3–5 septate.

### 2.4. Molecular Identification of Fusarium Strain

Based on its aggressive virulence, isolate #1 was chosen for further genetic identification based on the sequence of their internal transcribed spacer (ITS) region (Figure 2f). Briefly, the Nucleotide–Nucleotide BLAST (BLASTn) algorithm against the most recent available data in GenBank and the national center for biotechnology information website (NCBI, http://www.ncbi.nlm.nih.gov/gene/; accessed on 2 September 2022) and an evolutionary analysis revealed that the query sequence showed high similarity (query cover = 98%, E value = 0.0, and percent identity = 99.82%) with *Fusarium* equiseti strain CB33-2 (GenBank Accession No. MT558602.1; 561 bp) (Figure 2f). The new sequence of the small subunit ribosomal RNA gene (partial sequence), internal transcribed spacer 1 (ITS1), 5.8S ribosomal RNA gene and internal transcribed spacer 2 (ITS2; complete sequence), and large subunit ribosomal RNA gene (partial sequence) of isolate #1 was deposited to the NCBI GenBank under the name *F. equiseti* isolate YN-2022 (GenBank accession No. OP339844; 557 bp).

### 2.5. Antifungal Activity of DMAPAPP and Its Ru-DMAPAPP

**Figure 3:**
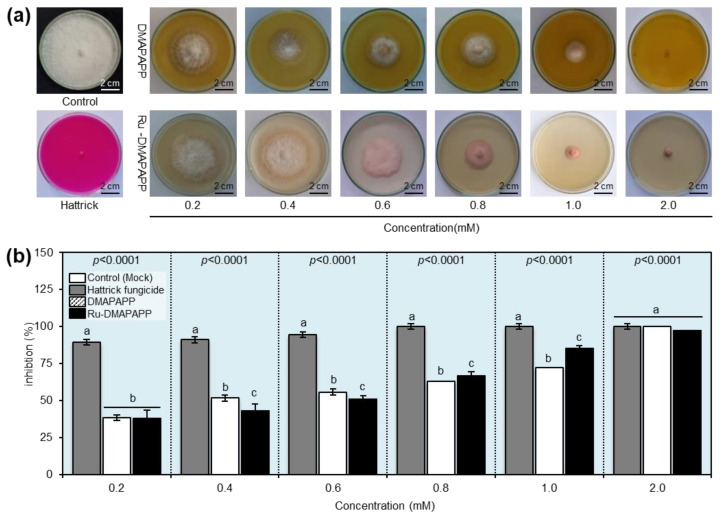
In vitro antifungal activity of two bis-chalcone derivatives, DMAPAPP and Ru-DMAPAPP, against *Fusarium equiseti*. (**a**) Antifungal activity of DMAPAPP and Ru-DMAPAPP against *F. equiseti*. (**b**) Inhibition (%) of mycelial radial growth of *F. equiseti* after treatment with different concentrations (0.2, 0.4, 0.6, 0.8, 1.0, and 2.0 mM) of DMAPAPP and Ru-DMAPAPP. Values represent the means ± standard deviations (mean ± SD). Different letters indicate statistically significant differences between treatments using the Tukey–Kramer honestly significant difference test (Tukey HSD; *p* < 0.05).

The antifungal activity of DMAPAPP and Ru-DMAPAPP was investigated at different concentrations (0.2, 0.4, 0.6, 0.8, 1.0, and 2.0 mM) against *F. equiseti* in vitro. The results revealed that all concentrations of the tested compounds suppressed the mycelial radial growth of *F. equiseti* as the concentration increased (Figure 3a). DMAPAPP was the most efficient molecule at the highest concentration (2 mM), completely inhibiting mycelial growth (100.00% ± 0.00), followed by Ru-DMAPAPP (97.14 ± 1.13%) (Figure 3b). It is worth mentioning that the susceptibility of *F. equiseti* to DMAPAPP at 2 mM was equivalent to the positive control “Hattrick” at the recommended dose (1 cm/L) without any significant difference between them.

### 2.6. Effects of DMAPAPP and Ru-DMAPAPP on the Development of Pepper Fusarium Wilt Disease under Greenhouse Conditions

**Figure 4:**
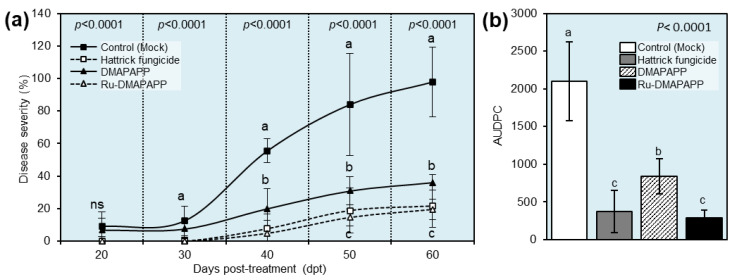
Effect of two bis-chalcone derivatives, DMAPAPP and Ru-DMAPAPP, on the development of pepper Fusarium wilt disease caused by *Fusarium equiseti* under greenhouse conditions. (**a**) Disease progress curves of fusarium wilt disease on pepper plants after treatment with DMAPAPP and Ru-DMAPAPP. (**b**) The area under the disease progress curve (AUDPC) of fusarium wilt disease on pepper plants after the treatment with DMAPAPP and Ru-DMAPAPP. Values represent the means ± standard deviations. Different letters indicate statistically significant differences between treatments using the Tukey–Kramer honestly significant difference test (Tukey HSD; *p* < 0.05).

The efficacy of DMAPAPP and Ru-DMAPAPP against *F. equiseti*, which causes wilt disease, was assessed under greenhouse conditions and compared to the positive control “Hattrick”. When compared to the control treatment, all tested compounds showed a significant reduction in disease severity (%) (Figure 4a). In comparison with both the negative (mock-treated) and positive control (Hattrick), Ru-DMAPAPP was the most effective compound and had the lowest disease severity (%) throughout the experiment (4.86 ± 5.57, 14.58 ± 3.76, and 19.44 ± 8.21%) at 40, 50, and, 60 days post-treatment (dpt), respectively (Figure 4a). Furthermore, treatment with a DMAPAPP or its complex resulted in a significant reduction in the area under the disease progression curve (AUDPC) (Figure 4b). Although the mock-treated control had the highest AUDPC value (2100.69 ± 522.67), Ru –DMAPAPP had the lowest AUDPC value (291.66 ± 97.31), which was not significantly different from the positive control (371.53 ± 276.65) (Figure 4b).

### 2.7. DMAPAPP and Ru-DMAPAPP Complex Alleviate the Oxidative Stress of F. equiseti-Infected Leaves

**Figure 5:**
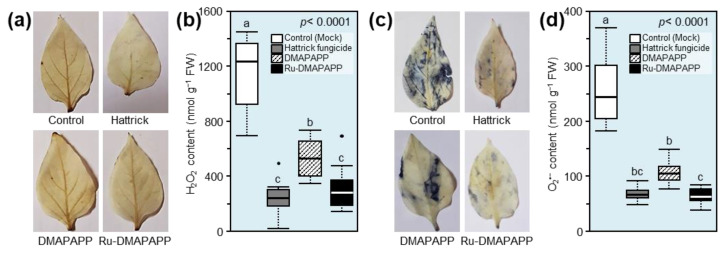
Effect of two bis-chalcone derivatives, DMAPAPP and Ru-DMAPAPP, on the oxidative stress associated with fusarium wilt disease caused by *Fusarium equiseti* on pepper plants under greenhouse conditions. (**a**) In situ histochemical visualization of superoxide anion (O_2_*^•^*^−^) using NBT-based staining at 72 h post-treatment (hpt) with DMAPAPP and Ru-DMAPAPP. (**b**) O_2_*^•^*^−^ content (nmol·g^−1^ FW) after the treatment with DMAPAPP and Ru-DMAPAPP. (**c**) In situ histochemical localization of hydrogen peroxide (H_2_O_2_) using DAB-based staining at 72 hpt with DMAPAPP and Ru-DMAPAPP. (**d**) H_2_O_2_ content (nmol·g^−1^ FW) after the treatment with DMAPAPP and Ru-DMAPAPP. Boxes show the interquartile ranges including values from 25 to 75%, horizontal thick lines indicate the medians, and whiskers reflect the highest and the lowest values of the data. Different letters indicate statistically significant differences between treatments using the Tukey–Kramer honestly significant difference test (Tukey HSD; *p* < 0.05).

Although the *F. equiseti*-infected leaves from mock-treated control exhibited an extensive accumulation of H_2_O_2_, as shown by the DAB-based in situ histochemical localization of H_2_O_2_ (Figure 5a), all tested compounds significantly reduced the H_2_O_2_ content within the *F. equiseti*-infected leaves, with a greater effect of Ru-DMAPAPP, which was notably comparable to the Hattrick-treated leaves (Figure 5b). Likewise, the NBT-based visualization of O_2_^•−^ in the mock-treated leaves (control) accumulated more blue color (an indicator of O_2_^•^^−^) (Figure 5c). However, all tested compounds notably decreased the O_2_^•^^−^ levels within the *F. equiseti*-infected leaves, with a greater effect of the Ru-DMAPAPP complex (Figure 5d). Taken together, these findings suggest that DMAPAPP and the Ru-DMAPAPP complex alleviate oxidative stress in *F. equiseti*-infected pepper leaves.

### 2.8. DMAPAPP and Ru-DMAPAPP Complex Enhanced the Profile of Total Soluble Phenolics and Flavonoids of F. equiseti-Infected Pepper Plants

**Figure 6:**
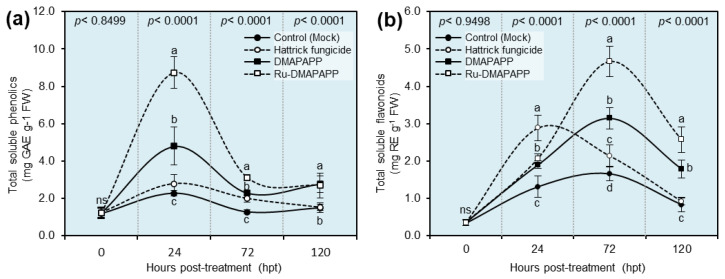
Effect of two bis-chalcone derivatives, DMAPAPP and Ru-DMAPAPP, on the non-enzymatic antioxidant defense compounds associated with fusarium wilt disease caused by *Fusarium equiseti* on pepper plants under greenhouse conditions. (**a**) Total soluble phenolics (mg GAE g^−1^ FW) and (**b**) total soluble flavonoids (mg RE g^−1^ FW). Values represent the means ± standard deviations (means ± SD). Different letters indicate statistically significant differences between treatments using the Tukey–Kramer honestly significant difference test (Tukey HSD; *p* < 0.05).

The total soluble phenolics of Ru-DMAPAPP-treated leaves reached their highest peak during the first 24 h post-treatment (hpt) (8.723 ± 0.852 mg GAE g^−1^ FW), followed by DMAPAPP-treated leaves (4.793 ± 1.017 mg GAE g^−1^ FW; Figure 6a), then fell again at 72 and 120 hpt, whereas no significant differences were noticed between mock-treated (negative control) and Hattrick-treated (positive control) leaves at all-time points (Figure 6a). On the other hand, the total soluble flavonoid content reached its highest peak at 72 hpt when the plants were treated with Ru-DMAPAPP (4.672 ± 0.406 mg RE g^−1^ FW) or DMAPAPP (3.150 ± 0.287 mg RE g^−1^ FW) and dropped again at 120 hpt (2.570 ± 0.343 and 1.786 ± 0.240 mg RE g^−1^ FW, respectively; Figure 6b).

### 2.9. Effects of DMAPAPP and Ru-DMAPAPP Complex on the Activity of Antioxidant Defense-Related Enzymes

**Figure 7:**
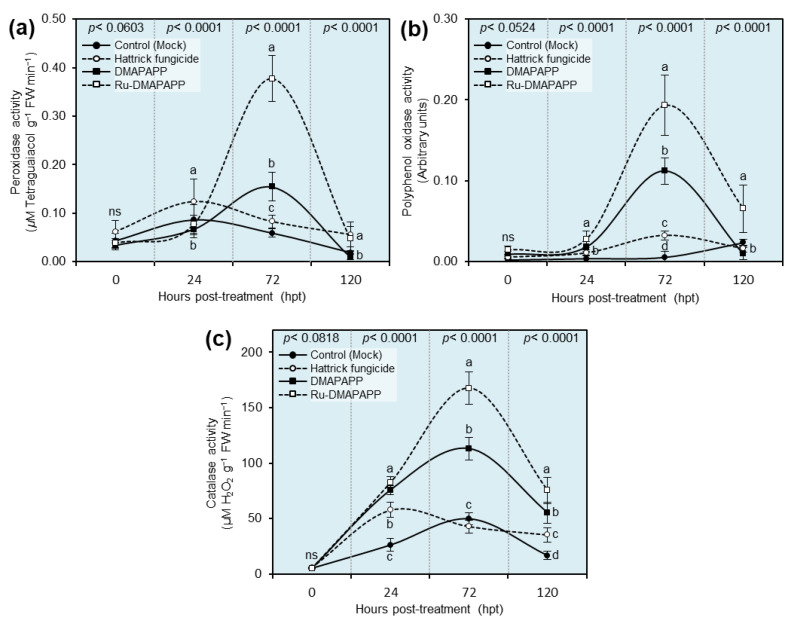
Effect of two bis-chalcone derivatives, DMAPAPP or Ru-DMAPAPP, on the enzymatic activity of the major antioxidant defense-related enzymes associated with fusarium wilt disease caused by *Fusarium equiseti* on pepper plants under greenhouse conditions. (**a**) Peroxidase activity (μM tetraguaiacol g^−1^ FW min^−1^), (**b**) polyphenol oxidase activity (arbitrary units), and (**c**) catalase activity (μM H_2_O_2_ g^−1^ FW min^−1^). Values represent the means ± standard deviations (means ± SD). Different letters indicate statistically significant differences between treatments using the Tukey–Kramer honestly significant difference test (Tukey HSD; *p* < 0.05).

The activity of three antioxidant defense-related enzymes, including peroxidase (POX; Figure 7a), polyphenol oxidase (PPO; Figure 7b), and catalase (CAT; Figure 7c), in *F. equiseti*-infected pepper leaves was evaluated at 0, 24, 72, and 120 hpt with DMAPAPP or the Ru-DMAPAPP complex (Figure 7). Generally, the highest enzymatic activity of all three tested enzymes peaked at 72 hpt when plants were treated with DMAPAPP or Ru-DMAPAPP, with superiority for the latter treatment. It is worth mentioning that POX activity was slightly elevated in Hattrick-treated leaves (0.124 ± 0.047 μM tetraguaiacol g^−1^ FW min^−1^) at 24 hpt. However, it was dramatically increased by treatment with the compound Ru-DMAPAPP at 72 hpt (0.377 ± 0.047 μM tetraguaiacol g^−1^ FW min^−1^), followed by DMAPAPP (0.154 ± 0.030 μM tetraguaiacol g^−1^ FW min^−1^) at the same time point (Figure 7a). Moreover, there was no significant difference between Hattrick and Ru-DMAPAPP treatment at 120 hpt (Figure 7a).

Likewise, the enzymatic activity of PPO was dramatically increased at 72 hpt with DMAPAPP (0.112 ± 0.016 arbitrary units) or Ru-DMAPAPP (0.193 ± 0.037 arbitrary units), then decreased at 120 hpt. Nevertheless, Ru-DMAPAPP-treated leaves showed significantly higher PPO activity (0.065 ± 0.029 arbitrary units) than other treatments at 120 hpt (Figure 7b). Similarly, the treatment of pepper plants with DMAPAPP or Ru-DMAPAPP led to an increase in the enzymatic activity of the CAT enzyme (75.779 ± 4.078 and 82.464 ± 5.136 µM H_2_O_2_ g^−1^ FW min^−1^, respectively) followed by Hattrick-treated leaves (57.637 ± 6.847 µM H_2_O_2_ g^−1^ FW min^−1^) after 24 hpt compared to the control (26.161 ± 5.878 µM H_2_O_2_ g^−1^ FW min^−1^) (Figure 7c). However, CAT activity was also dramatically increased at 72 hpt with Ru-DMAPAPP (167.617 ± 14.648 µM H_2_O_2_ g^−1^ FW min^−1^) or DMAPAPP alone (113.127 ± 10.351 µM H_2_O_2_ g^−1^ FW min^−1^).

### 2.10. DMAPAPP and Ru-DMAPAPP Stimulate the Expression of Antioxidant Enzymes

**Figure 8:**
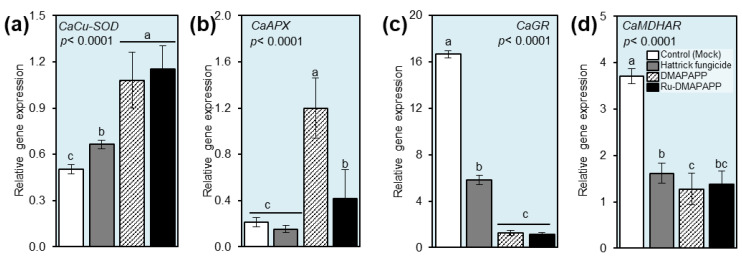
Effect of two bis-chalcone derivatives, DMAPAPP and Ru-DMAPAPP, on gene expression of major antioxidant defense-related enzymes associated with fusarium wilt disease caused by *Fusarium equiseti* on pepper plants under greenhouse conditions. (**a**) Relative gene expression of superoxide dismutase [Cu-Zn] 1 (*CaCuSOD*), (**b**) relative gene expression of cytosolic ascorbate peroxidase 1 (*CaAPX*), (**c**) relative gene expression of glutathione reductase, chloroplastic (*CaGR*), and (**d**) relative gene expression of monodehydroascorbate reductase (*CaMDHAR*). Values represent the means ± standard deviations (means ± SD). Different letters indicate statistically significant differences between treatments using the Tukey–Kramer honestly significant difference test (Tukey HSD; *p* < 0.05).

The gene expression of four antioxidant enzymes, including superoxide dismutase [Cu-Zn] 1 (*CaCuSOD*; Figure 8a), cytosolic ascorbate peroxidase 1 (*CaAPX*; Figure 8b), glutathione reductase, chloroplastic (*CaGR*; Figure 8c), and monodehydroascorbate reductase (*CaMDHAR*; Figure 8d) were investigated at 72 hpt. The results revealed that plants treated with DMAPAPP and Ru-DMAPAPP significantly upregulated *CaCuSOD* and *CaAPX* compared with both controls. Nevertheless, DMAPAPP and Ru-DMAPAPP significantly decreased the expression levels of *CaGR* and *CaMDHAR*.

### 2.11. Effect of DMAPAPP and Ru-DMAPAPP on the Growth Parameters and Total Chlorophyll in F. equiseti-Infected Pepper Plants

The effect(s) of DMAPAPP and Ru-DMAPAPP on the growth of *F. equiseti*-infected pepper plants (as expressed by plant height (cm plant^−1^), the number of leaves per plant, shoot fresh weight (g plant^−1^), and total leaf area) as well as the photosynthetic pigments (as expressed by total chlorophyll) were recorded at 60 days after transplanting (Table 1). Although there were no significant differences between the mock control (26.91 ± 4.77 cm) and DMAPAPP-(27.41 ± 2.02) or Ru-DMAPAPP-treated (30.58 ± 3.55) plants in terms of plant height, treatment with the Ru-DMAPAPP complex significantly increased the number of leaves per plant (29.00 ± 2.21 leaves per plant), shoot fresh weight (14.39 ± 3.57 g plant^−1^), and total leaf area (12.38 ± 3.72 cm^2^). Moreover, the total chlorophyll content of pepper leaves significantly increased as a result of treatment with the Ru-DMAPAPP complex (81.09 ± 2.67) or DMAPAPP alone (72.24 ± 7.27) compared with mock-treated plants (38.28 ± 13.84; Table 1).

## 3. Discussion

Like many other solanaceous crops, sweet pepper is susceptible to Fusarium wilt disease, caused by *F. equiseti*, which is the most destructive disease of many solanaceous crops, such as potato, tomato, pepper, and eggplant, and causes significant crop losses worldwide. In nature, *F. equiseti* is a soil-borne phytopathogen and invades the vascular system of infected plants internally. Although several physical, cultural, and biological management strategies have been utilized to combat this destructive disease [29,30], chemical fungicides have mainly and conventionally been used to control this disease. Chemical fungicides are relatively expensive and pose many risks to the environment, humans, animals, plants, and non-target microorganisms, such as beneficial soil microflora. Nevertheless, none of these strategies has been able to entirely suppress the disease due to the broad host range and the sustained survival of its causal agent in the soil. Therefore, searching for effective alternatives to conventional fungicides is required for sustainable management strategies for Fusarium wilt disease of sweet pepper and other Solanaceae crops. These alternative strategies include the synthesis and implementation of sustainable ecofriendly natural compounds, such as chalcone derivatives, to reduce the usage of fungicides entirely or at least partially. Additionally, we believe that it is better to safeguard the entrance point of the phytopathogenic fungus to the host plant rather than changing the entire soil microflora community.

In the current study, we tested the potential application of two bis-chalcone derivatives, including DMAPAPP, which was previously synthesized by Gaber et al. [27], and its metal complex with ruthenium, Ru-DMAPAPP, which was recently prepared by El-Sayed et al. [28] to protect the roots of sweet pepper against the Fusarium wilt disease. Our in vitro findings demonstrated that both compounds showed a strong dose-dependent fungistatic activity against *F. equiseti* and completely inhibited mycelial growth at concentrations as low as 2 mM, which was comparable to the most commonly used fungicide, “Hattrick”, at the recommended dose (1 cm L^−1^). Moreover, our greenhouse experiments showed that both tested compounds significantly reduced the disease development as expressed by lower disease severity (%) as well the as lower area under the disease progression curve (AUDPC), with superiority for the Ru(III) complex with a chalcone ligand.

Previously, several chalcone derivatives showed strong antifungal activities against numerous phytopathogenic fungi, such as *Helminthosporium maydis*, *Sclerotinia sclerotiorum*, *Rhizoctonia solani*, *Gibberella zeae,* and *Botrytis cinerea* [24], as well as the fungi-like agent, *Phytophthora infestans* [25]. In general, the biological activity of chalcones is recognized by the presence of reactive *α* and *β* unsaturated keto groups [31]. However, its antifungal activity depends on its capacity to interact with sulfhydryl groups on the fungal cell wall [32]. Moreover, chalcones inhibit the β (1,3)-glucan and chitin synthase enzymes that catalyze the biosynthesis of β (1,3)-glucan and chitin polymers of the fungal cell wall, respectively [33,34]. Collectively, we suggest that the antifungal activity of the chalcone ligand and its Ru(III) complex might be due to their direct effect on the cell wall of *F. equiseti*.

Moreover, our findings showed a significant increase in the antifungal activity of the Ru(III) complex (Ru-DMAPAPP) that was greater than that of the parent ligand (DMAPAPP). This phenomenon could be explained based on Tweedy’s chelation theory [35]. Briefly, the Ru(III) metal ion partially shares its positive charge with the donor group and the π-electron delocalization within the whole chelate ring system, which decreases its polarity but increases the lipophilic nature of the central metal atom. In other words, metal complexation improves the hydrophobicity and liposolubility of the complex, leading to better penetration through the cell membrane. Together, this might explain why Ru-DMAPAPP exhibited a relatively higher fungistatic activity against *F. equiseti*.

Moreover, although the severity of Fusarium wilt disease on the non-treated plants was increased progressively throughout the experiment, Ru-DMAPAPP and its parent ligand (DMAPAPP) efficaciously suppressed symptom development, reduced disease severity, and decreased the AUDPC of pepper plants at over 60 days post-treatment (dpt). Additionally, pepper plants that were soaked in DMAPAPP or Ru-DMAPAPP showed no phytotoxicity on the treated plants, as expressed by improved growth performance (plant height, the number of leaves, shoot fresh weight, total leaf area, and total chlorophyll). This might be due to their direct fungistatic activity against *F. equiseti* at the entrance point on pepper roots since the roots of pepper seedlings were soaked in DMAPAPP or Ru-DMAPAPP solutions before being planted in soil infested with *F. equiseti*. However, more investigations are required to better understand the positive effects of the chalcone ligand and its metal complexes on the treated plants.

Another possible mechanism of the potential role(s) of bis-chalcones in *F. equiseti*-infected pepper plants involves multifaceted antioxidant defense machinery. To better understand the potential antioxidant role(s) of DMAPAPP or Ru-DMAPAPP in treated plants, we first tested their radical scavenging activity via an examination of their antioxidant abilities by DPPH in vitro. Both synthetic chalcone derivatives were found to be strongly reactive towards DPPH radicals and showed considerable reducing abilities, with superiority to Ru-DMAPAPP with a chalcone ligand. In agreement with these findings, several chalcone derivatives were reported previously as strong antioxidant agents [36,37,38]. Moreover, it has been previously reported that chalcones, after metal complexation, showed significant scavenging activities, particularly in the case of nickel metal in DPPH radical scavenging (up to 75%), indicating their strong antioxidant power, whereas the parent ligand showed low significant scavenging activities (less than 25%), demonstrating their weak antioxidant power [22]. Taken together, these findings suggest that, although both DMAPAPP and Ru-DMAPAPP can be considered potential antioxidant compounds, metal complexation increases their scavenging activity.

Due to the strong antioxidant activities of DMAPAPP and Ru-DMAPAPP in vitro, we suggested that they might stimulate the antioxidant defense machinery of treated plants. Interestingly, the in situ histochemical localization of reactive oxygen species (ROS), particularly hydrogen peroxide (H_2_O_2_) and superoxide anion (O_2_^•−^), showed that infection with *F. equiseti* induced an extensive accumulation of H_2_O_2_ and O_2_^•−^, which generated strong oxidative stress in infected pepper leaves. However, treatment with DMAPAPP or Ru-DMAPAPP significantly reduced the accumulation of both free radicals (H_2_O_2_ and O_2_^•−^) within *F. equiseti*-infected leaves, with a greater effect of Ru-DMAPAPP, which was notably comparable to the Hattrick-treated leaves. Together, these findings support our hypothesis that bis-chalcone derivatives elicit the activation of a multilayered antioxidative system to neutralize the harmful effect of ROS within *F. equiseti*-infected leaves.

In general, the antioxidant defense machinery of higher plants involves two major mechanisms [39,40,41,42]: (I) the enzymatic antioxidants, also known as the front antioxidant defense line, mainly rely on a variety of scavenging enzymes, including ascorbate peroxidase (APX), catalase (CAT), glutathione peroxidase (GPX), glutathione reductase (GR), monodehydroascorbate reductase (MDHAR), polyphenol oxidase (PPO), peroxidase (POX), and superoxide dismutase (SOD) [40,41], and (II) non-enzymatic antioxidants, also known as the second antioxidant defense line, mainly rely on hydrophilic metabolites, such as phenolics and flavonoids, and lipophilic antioxidants, such as carotenoids [40,43,44,45,46].

Interestingly, our findings showed that treating pepper plants with DMAPAPP or RU-DMAPAPP notably increased the enzymatic activity of peroxidase (POX), polyphenol oxidase (PPO), and catalase (CAT), which was associated with a significant decrease in ROS levels, particularly H_2_O_2_ and O_2_^•−^. Likewise, bis-chalcone derivatives enhanced the transcript levels of superoxide dismutase [Cu-Zn] (*CaSOD-Cu*), L-ascorbate peroxidase 1, and cytosolic (*CaAPX1*) but not glutathione reductase, chloroplastic (*CaGR1*), and monodehydroascorbate reductase (*CaMDHAR*). SOD catalyzes the conversion of O_2_^•−^ to H_2_O_2_. Subsequently, APX catalyzes the transformation of H_2_O_2_ into H_2_O while using ascorbate as an electron source [47]. Additionally, POD is an important stress-associated enzyme that is known to control the level of H_2_O_2_ in plant tissues [44]. This enzyme directly scavenges H_2_O_2_ and O_2_^•−^ and reduces their reactivity [43]. Collectively, our findings suggest that the application of DMAPAPP or its metal complex, RU-DMAPAPP, might be involved in the activation of enzymatic antioxidant defense machinery to maintain the homeostasis of ROS within infected plants via the induction of APX, POD, SOD, and other antioxidant enzymes.

Moreover, our findings showed that DMAPAPP or Ru-DMAPAPP boosted the endogenous contents of total soluble phenolics to reach their highest peak during the first 24 h post-treatment (hpt). It has been reported that the cellular antioxidant capacity in higher plants is associated with the total phenolic content [48]. Likewise, bis-chalcone derivatives heightened the profile of the total soluble flavonoids to reach their highest peak at 72 hpt. The induction of total soluble phenolics and total soluble flavonoids was negatively correlated with disease progression and might directly inhibit the colonization of *F. equiseti*. Together, these findings suggest that the application of bis-chalcone derivatives might be involved in the activation of non-enzymatic antioxidant defense machinery to suppress the progression of Fusarium wilt disease via the induction of total soluble phenolics and total soluble flavonoids.

In summary, bis-chalcone derivatives (DMAPAPP or Ru-DMAPAPP) diminish the damaging effect of *F. equiseti* on sweet pepper plants via a complex multilayered defense system that includes at least three major mechanisms: (i) DMAPAPP and Ru-DMAPAPP have strong dose-dependent antifungal activity against *F. equiseti*; (ii) DMAPAPP and Ru-DMAPAPP are involved in the activation of enzymatic antioxidant defense machinery to maintain the homeostasis of ROS within *F. equiseti*-infected plants via the induction of APX, POD, SOD, and other antioxidant enzymes; and (iii) DMAPAPP and Ru-DMAPAPP are involved in the activation of non-enzymatic antioxidant defense machinery to suppress the progression of Fusarium wilt disease via the induction of total soluble phenolics and total soluble flavonoids. However, more investigations are required to better understand the molecular connections and signaling between these mechanisms.

## 4. Materials and Methods

### 4.1. Tested Compounds

In the current study, we tested the potential application of two bis-chalcone derivatives, including 3-(4-dimethyl amino-phenyl)-1-{6-[3-(4 dimethyl amino-phenyl)-a cryloyl]-pyridin-2-yl}-propanone (DMAPAPP), which was previously synthesized by Gaber et al. [27], and its metal complex with ruthenium III (Ru-DMAPAPP), which was prepared previously by El-Sayed et al. [28] (Figure 1a), against *F. equiseti*, the causal agent of Fusarium wilt disease. In addition, the commonly used Hattrick fungicide (Tebuconazole 6% FS; (RS)-1-p-chlorophenyl 4,4-dimethyl-3-(1H-1,2,4-triazol—1-methyl)pentan-3-ol) at the recommended dose (1 cm^3^ L^−1^ water) was used as a positive control. The tested compounds were first dissolved in 5 mL of 100% dimethylformamide (DMF) and then adjusted to a final volume of 100 mL using sterilized water to make a 5 mM stock solution that was diluted and used in all subsequent experiments.

### 4.2. Antioxidant and Radical Scavenging Assay

The free radical scavenging activity of DMAPAPP and Ru-DMAPAPP, in addition to Hattrick fungicide, was spectrophotometrically determined using 2,2-diphenyl-1-picrylhydrazyl (DPPH) as described by Reddy et al. [49] with slight modification as described by Hijaz et al. [50]. Briefly, 100 µL of seven concentrations of DMAPAPP or Ru-DMAPAPP (0.03, 0.06, 0.13, 0.25, 0.50, 0.75, or 1.00 mM) was mixed with 900 µL of a 0.1 mM DPPH methanolic solution. Five replicates (*n* = 5) were tested for each concentration. Subsequently, the mixture was incubated at room temperature for 30 min. Then, the absorbance of DPPH free radicals was measured at 517 nm. Methanol was used as a blank. The IC_50_ was defined as the amount of the extract required to decrease the absorbance of the blank to one half. The radical scavenging activity (%) was calculated using Equation (1):

(1) $$\mathrm{Scavenging}\;\mathrm{activity}\;\left(\%\right)=\frac{A_{0}-A_{1}}{A_{0}}\times 100$$

where A_0_ is the absorbance of the control (blank) and A_1_ is the absorbance of DMAPAPP or Ru-DMAPAPP. The experiment was repeated three times at each concentration.

### 4.3. Isolation, Morphological Characterization, Molecular Identification of Fusarium Isolates

#### 4.3.1. Pathogen Isolation

Three Fusarium isolates were isolated from diseased pepper plants exhibiting typical symptoms of wilt and crown and root rot diseases using the hyphal tip technique. Briefly, plant samples were collected, mainly from Tanta, EL-Gharbiya Governorate, Egypt (Latitude: 30°47′18.49″ N and Longitude: 31°00′6.91″ E). The roots, crowns, and stems were first cut into small pieces, cleaned with distilled water, disinfected with sodium hypochlorite (2%) for 3 min, rinsed again with sterile distilled water to eliminate any remaining bleach water, and then dried using sterile filter papers. The shards were then sliced lengthwise and placed on Petri dishes with PDA medium. The medium was spiked with 200 mg L^−1^ Streptomycin to prevent bacterial growth. The plates were incubated at 27 ± 2 °C for 4 to 5 days. Subsequently, the fungus was purified using the single-spore technique and maintained on PDA. The obtained pure cultures were maintained by repeated subculturing at 45-day intervals for further studies. The fungal isolates that might be associated with the Fusarium wilt disease were first characterized based on their cultural, morphological, and macroscopic characteristics, then molecularly identified based on the sequence of their internal transcribed spacer (ITS) region.

#### 4.3.2. Morphological and Cultural Characterization of *Fusarium* sp. Isolates

In the beginning, cultural characteristics such as the diameter, color, reverse color, texture, and the presence of sporodochia of the colony were visually noted. Additionally, the morphological characteristics of *Fusarium* sp. isolates were identified according to Hami et al. [15]. Briefly, spores were collected from 10-day-old colonies grown on PDA using a paintbrush, suspended in a sterilized 0.2% agar solution (containing 0.05% Tween 80), then filtered through cheesecloth to remove the fungal mycelia. The spore suspension of each isolate was used to prepare semipermanent slides after staining with lactophenol cotton blue for microscopy assays. The studied morphological characteristics included hyphae characteristics (septation, width, and color), conidiophore and conidia characteristics (shape, size, and color), and chlamydospore characteristics (shape, size, and color) [15].

#### 4.3.3. Molecular Identification of *Fusarium* sp. Isolates

For molecular identification, the most aggressive isolate of *Fusarium* sp. was cultured on sterile potato dextrose broth and maintained at 27 ± 2 °C for 5 days. Later, fungal mycelium was harvested, filtered through cheesecloth, washed twice with sterile water, and finally dried using sterile filter paper as described above. Subsequently, approximately 0.1 g of fungal mycelium was ground to a fine powder using liquid nitrogen. Genomic DNA was extracted and purified, and the targeted sequences of the ITS region were PCR-amplified according to Atallah and Yassin [51] and Atallah et al. [52]. For sequencing, the PCR product was purified using a Qiagen Gel extraction kit and sent to the AuGCT sequencing facility (Aoke Dingsheng Biotechnology Co., Beijing, China). The bidirectional sequencing of the ITS-5.8S rDNA sequence was performed using Sanger sequencing. Consensus sequences were determined and assembled using DNAMAN X software (Lynnon Corp., Quebec, Canada) and compared to the most recent available data in GenBank and the national center for biotechnology information website (NCBI, http://www.ncbi.nlm.nih.gov/gene/; accessed on 2 September 2022) using the Nucleotide–Nucleotide BLAST (BLASTn) algorithm. The assembled sequence was deposited to the NCBI GenBank.

#### 4.3.4. Phylogenetic Analysis

A phylogenetic reconstruction was performed using bidirectional sequences of the ITS-5.8S rDNA sequence. About 25 reference strains/isolates were selected and used for multiple sequence alignment using ClustalW multiple sequence alignment algorithms. Sequence alignments were manually edited and trimmed when needed. Finally, the phylogenetic evolutionary tree was inferred using the maximum-likelihood algorithm with 500 bootstrap replications using MEGA X software.

### 4.4. Pathogenicity Test

The pathogenicity test was carried out on healthy pepper seedlings of the cultivar Top star (30 days old). In a sand–barley medium (75 g of barley grains + 25 g of sand + 100 mL of water), three isolates of the Fusarium wilt pathogen were multiplied and maintained separately. Four discs of each isolate were evenly inoculated into the sand–barley medium and cultured at 27 ± 2 °C for two weeks. The pathogenicity of the three isolates was tested by artificially inoculating the sand–barley culture in the soil at 5% (*w*/*w*). The pots (30 cm in diameter) were filled with infected soil. Five seedlings (30 days) were transplanted into pots (30 cm). The observation of disease incidence was monitored regularly for symptom expression and pathogenicity. The disease incidence was assessed using Equation (2):

(2) $$\mathrm{Disease}\;\mathrm{Incidence}\;(\%)=\frac{Number\;of\;infected\;plants}{Total\;number\;of\;plants}*100$$

The disease severity was graded on a 0–4 scale, with zero indicating no infection and four indicating entirely infected plants. The 0–4 scale of disease severity was classified as follows: (0: no symptomatic leaves; 1: mild infection, around 25% of full scale (one or two leaves became yellow); 2: moderate infection, two or three leaves have turned yellow and half of the leaves have begun to wilt; 3: severe infection, all of the plant’s leaves turned yellow, with 75% of them wilting, and growth was slowed; and 4: complete infection, in which all of the plant’s leaves turned yellow, all of the leaves wilted, and the plant died) [53]. The DS% was calculated 20, 40, and 60 days post-inoculation according to Equation (3):

(3) $$\mathrm{Disease}\;\mathrm{severity}\;\left(\%\right)=\frac{\sum \left(\mathrm{Scale}\times \;\mathrm{Number}\;\mathrm{of}\;\mathrm{plants}\;\mathrm{infected}\right)}{\left(\mathrm{highest}\;\mathrm{scale}\;\times \mathrm{total}\;\mathrm{number}\;\mathrm{of}\;\mathrm{plants}\right)}\times 100$$

### 4.5. Antifungal Activity

The antifungal activity of DMAPAPP and Ru-DMAPAPP was assessed in vitro against the pathogenic fungus *F. equiseti* using the agar diffusion method [54]. Six serial dilutions of each compound were prepared by mixing the proper volumes of each compound with 20 mL of potato dextrose agar medium to create six final concentrations of 0.2, 0.4, 0.6, 0.8, 1, and 2 mM. Sterilized DMF was used as a negative control at a final concentration of 0.5 percent in the PDA medium. Following that, a 0.5 cm diameter mycelial plug from a freshly prepared *F. equiseti* culture was placed on the surface of the premade Petri dishes, and the dishes were incubated at 27 ± 2 °C for seven days until the mycelial growth covered the entire control plate. Each Petri dish’s mycelium growth (cm) was measured after 7 days. The experiment had six biological replicates and used a completely randomized design. The percentage of growth inhibition was determined using Equation (4):

(4) $$\mathrm{Inhibition}\;(\%)=\frac{C\;-T}{C}\times 100$$

where “C” stands for the mycelium growth in the negative control plate and “T” stands for mycelium growth in the treatment plate. The experiment was repeated twice.

### 4.6. Greenhouse Experiment, Disease Assessment, and Growth Parameters

Two pot experiments were conducted in pots to evaluate the effects of the tested compounds on pepper plants infected with a pathogenic fungus at the Agricultural Faculty Farm, Tanta, Egypt, during the spring seasons of 2020 and 2021. The most aggressive isolate of *F. equiseti*, a pathogenic fungus, was cultivated and maintained for two weeks at 27 ± 2 °C on sand–barley medium in 500 mL glass bottles. Plastic pots (30 cm in diameter) containing sterilized sandy clay soil (1:1 *w*/*w*) were infested with a fresh inoculum of fungus. The inoculum of fungus was inoculated into plastic pots (30 cm in diameter) containing sterilized sandy clay soil (1:1 *w*/*w*) at a rate of 5% (*w*/*w*) potential inoculum, and the fungal inoculum was combined with the sterilized soil surface of each pot. The pots were watered and stored in a greenhouse (28 ± 2 °C, 70% relative humidity, and 16 h photoperiod during the experimental period) for ten days before transplanting pepper seedlings. Then, for 2 h, a 30-day-old Top star pepper seedling cultivar was soaked in each treatment’s solution before being planted in pots with soil infested with *F. equiseti*. Without the inclusion of the tested substances, a mock treatment was made. In two different investigations, six biological replications were kept for each treatment. Every week, the plants were watered weekly, and agricultural operations and fertilization were carried out as recommended. At 20, 40, and 60 days after transplanting, the degree of wilting (disease severity) was measured. A complete randomized block design was used, with six biological replications per treatment and three plants for each replicate. As previously stated, the severity of wilt disease was assessed according to [53]. In both the 2020 and 2021 seasons, 20 plants were randomly selected from each treatment at the end of the growing season to determine some growth characteristics, such as the plant height (cm), the number of leaves per plant, the total leaf area, the fresh weight (g plant^−1^), the total chlorophyll, the total leaf area, and the fresh weight (g/plant).

### 4.7. In Situ Histochemical Localization of Hydrogen Peroxide (H_2_O_2_) and Superoxide Anion (O_2_^•−^)

Hydrogen peroxide (H_2_O_2_) was histochemically localized using 3,3-diaminobenzidine (DAB; Sigma–Aldrich, Darmstadt, Germany) as described by [55,56] with slight modifications as described by [57,58]. Briefly, three leaves were collected from each plant from three different positions (top, middle, and lower sections), directly immersed and vacuum-infiltrated with 1 mg mL^−1^ DAB in 10 mM 2-(*N*-morpholino)ethanesulfonic acid (MES) buffer (pH 6.5), and incubated at room temperature under light for 8 h until the development of a brown color. After the illumination, leaves were bleached in 0.15% (*w*/*v*) trichloroacetic acid (TCA) in 4:1 (*v*/*v*) ethanol/chloroform imaged using a Chemi Imager 4000 digital imaging system (Alpha Innotech Corp., San Leandro, CA, USA), and the intensity of the brown color was quantified using the Image J image processing program (Fiji version; http://fiji.sc; accessed on 8 May 2022) [59]. Likewise, the in situ histochemical localization of superoxide anion (O_2_^•−^) was assessed using the nitro blue tetrazolium (NBT; Sigma–Aldrich, Darmstadt, Germany). As described above, three leaves were collected, immersed, and vacuum-infiltrated in 1 mg mL^−1^ NBT in 50 mM potassium phosphate buffer (pH 6.4) then incubated under light for 30 min at room temperature until the development of a purple color. Subsequently, leaves were bleached and imaged, and the intensity of the purple color was quantified as described above.

### 4.8. Total Soluble Phenolic and Total Flavonoid Compounds

Folin–Ciocalteu reagent (FCR) was used to measure the total soluble phenolic compounds as previously described [60] with slight modification. In a nutshell, phenolics were extracted from 100 mg of fresh pepper leaves for 24 h using 20 mL of 80% methanol. After that, 1 mL of FCR (10%) was added to 0.2 mL of fresh pepper leaf methanolic extract and vortexed for 30 s, and 0.8 mL of 7.5 percent sodium carbonates (*w*/*v*) were added to the mixture three minutes later. The mixture was then incubated for 30 min at room temperature, and the absorbance was measured at 765 nm. The gallic acid equivalents per gram of fresh weight (mg GAE g^−1^ FW) were used to calculate the total soluble phenolic content.

Furthermore, the total soluble flavonoids were determined according to the method in [61]. In a nutshell, 1 mL of methanolic pepper leaf extract was added to 1 mL of aluminum chloride (2 percent in methanol). Subsequently, the mixture was vigorously mixed, incubated for 15 min at room temperature, and measured for its absorbance at 430 nm. The amount of flavonoid in each gram of fresh weight was measured in mg of rutin equivalents per gram of fresh weight (mg RE g^−1^ FW).

### 4.9. Antioxidant Enzymatic Activity

Infected pepper leaves were taken at 1, 3, and 5 days after treatment for enzyme analysis. The guaiacol-dependent peroxidases (POX) and polyphenol oxidase (PPO) activities were colorimetrically determined at 25 °C using a UV-160 spectrophotometer (Shimadzu, Japan). Then, 0.5 g of pepper leaf tissues were homogenized in 3 mL of 50 mM Tris buffer (pH 7.8) with 1 mM EDTA-Na2 and 7.5 percent polyvinylpyrrolidone in a prefreeze mortar and pestle (0–4 °C). The homogenate was then centrifuged at 4 °C for 20 min at 12,000 rpm. The POX activity was assessed by measuring the formation of the guaiacol-bound product at 436 nm [62]. The reaction mixture included 100 µL of crude enzyme extract, 100 µL of guaiacol, 100 µL of 12 mM H_2_O_2_, and 2.2 mL of 100 mM sodium phosphate buffer (pH 6.0). Using an extinction coefficient of 26.6 mM^−1^ cm^−1^ for the conjugate, the increase in the absorbance at 436 nm (A_436_) was observed as the conjugate developed. The activity of polyphenol oxidase (PPO) was assessed using the method in [63]. A 3 mL buffered catechol solution (0.01 M), freshly produced in 0.1 M phosphate buffer, was added to the reaction mixture (pH 6.0). Then, 100 μL of crude enzyme extract was added to start the process. For 3 min, the changes in absorbance at 495 nm (A_495_) were recorded every 30 s. For CAT activity, 0.5 g of pepper leaf tissues were homogenized with 3 mL of 50 mM Tris buffer (pH 7.8) containing 1 mM EDTA-Na2 and 7.5% polyvinylpyrrolidone (PVP) then centrifuged at 11,269× *g* at 4 °C for 20 min. The CAT activity was determined according to [64]. The CAT activity was measured by following the decomposition of H_2_O_2_ at 240 nm and was expressed as µM H_2_O_2_ g^−1^ FW min^−1^.

### 4.10. Gene Expression Analysis

The expression levels of four antioxidant genes were analyzed in total RNA extracted from pepper leaves (72 h after treatment) using an RNA extraction kit (Simply P Total RNA Extraction kit, catalog No BSC52S1) according to the manufacturer’s protocol. Total RNA was extracted from 100 mg of frozen pepper tissue RNA samples that were treated with DNAse to remove the genomic DNA contamination. The purity and concentration of the total RNA were determined using a NanoDrop 2000 spectrophotometer (Thermo Scientific, USA). RNA was reverse-transcribed to cDNA using a TOP script™ cDNA Synthesis Kit According to the manufacturer’s protocol. The cDNA synthesis reaction mixture (20 μL) contained 1 μg of total RNA, 1 μL of 25 pM oligo dT primer, and RT premix, and the synthesis was carried out at 37 °C for 90 min quantitative real-time PCR (qPCR) assays. The sequences of the primers used for the RT-PCR analysis are shown in Table 2.

Primer specificity and amplification efficiency were also verified for each gene with a melting curve analysis (after 40 cycles) and agarose gel electrophoresis. The qPCR reaction was performed in a 25 μL reaction mixture containing 5 μL of diluted cDNA sample and 12.5 μL of TOPreal™ qPCR 2X PreMIX (SYBR Green with low ROX and each forward and reverse primer (0.4 μM)). The initial denaturation was performed at 95 °C for 10 min, followed by 40 cycles of denaturation at 95 °C for 20 s and annealing and extension at 60 °C for 45 s. The pepper actin gene was used as the internal control for the normalization of the expression of the target genes. The relative expression was calculated using the 2^−ΔΔCT^ method [65]. One housekeeping gene was used for the normalization of gene expression (*CaACTIN*).

### 4.11. Statistical Analyses

All experiments were repeated twice (with six biological replicates for each treatment) during two different growing seasons (2020 and 2021). All results were statistically examined using the analysis of variance technique (ANOVA), followed by the Tukey–Kramer honestly significant difference test (Tukey HSD, *p* ≤ 0.05) as a post hoc analysis for pairwise comparisons. Statistical analyses were made using JMP Data Analysis Software version 14, and the values were reported as the means of two experiments.

## 5. Conclusions

The present study indicated that DMAPAPP and Ru-DMAPAPP, as safe chemical elicitors, were very effective in the control of pepper Fusarium wilt disease by the reduction of disease assessments. The mechanism of action underlying the effect of DMAPAPP and Ru-DMAPAPP might be related to the direct antifungal activity against *F. equiseti*. Our results confirmed that the fungistatic properties of DMAPAPP and Ru-DMAPAPP mainly target the fungal cell membrane and its components by inhibiting *F. equiseti* growth. In addition, DMAPAPP and Ru-DMAPAPP strengthen the defense system in pepper leaves by stimulating the activity of defense-related enzymes, including POX, PPO, and CAT, and non-enzymatic antioxidant total soluble phenolics and flavonoids and upregulating the gene expression of the *Cu-SOD* and *AXP* genes.

## Figures and Tables

**Table 1:** Growth parameters on pepper plants 60 days after transplanting.

Treatment	Plant Height (cm plant^−1^)	Number of Leaves	Shoot Fresh Weight (g plant^−1^)	Total Leaf Area (cm^2^)	T. Chlorophyll (Spad)
Mock control	26.91 ± 4.77 ^a^	21.50 ± 4.25 ^b^	6.207 ± 1.14 ^c^	07.03 ± 1.20 ^b^	38.28 ± 13.84 ^c^
Hattrick fungicide	21.75 ± 2.83 ^b^	20.25 ± 2.22 ^b^	6.238 ± 1.02 ^c^	05.38 ± 0.76 ^b^	65.35 ± 5.06 ^b^
DMAPAPP	27.41 ± 2.02 ^a^	18.50 ± 4.23 ^b^	11.57 ± 1.09 ^b^	05.17 ± 0.62 ^b^	72.24 ± 7.27 ^ab^
Ru-DMAPAPP	30.58 ± 3.55 ^a^	29.00 ± 2.21 ^a^	14.39 ± 3.57 ^a^	12.38 ± 3.72 ^a^	81.09 ± 2.67 ^a^

Values shown are the averages and standard deviations (means ± SD). Different letters indicate statistically significant differences among treatments, according to Tukey’s honestly significant difference test (*p* < 0.05).

**Table 2:** Primers used for gene expression analysis of antioxidant-related genes of pepper (*Capsicum annuum*) using real-time RT-PCR.

**Description**	**Gene**	**NCBI** **Accession Number**		**Primer** **(Forward and Reverse)**
Superoxide dismutase [Cu-Zn]	*CaSOD-Cu*	NM_001398340.1	F: R:	TACCACAAATGGCTGCATGT TTTGCTGAGCTCATGTCCAC
L-ascorbate peroxidase 1, cytosolic	*CaAPX1*	NM_001325037.1	F: R:	TAGGGAGCAGTTTCCCACAC AACACGTCCCTCAAGTGGTC
Glutathione reductase, chloroplastic	*CaGR1*	XM_016710630.2	F: R:	GGTGGAGGGTACATTGCTGT TGCCACCTTCTTCTGCTTTT
monodehydroascorbate reductase	*CaMDHAR*	XM_016687442.2	F: R:	AGATCGTTGGTGCATTCCTC ATCAACCAGGCACGAAAAAC
Actin (Housekeeping gene)	*CaACTIN*	XM_016722297.2	F: R:	CCTCGTCACACGGGAGTAAT CACGATTAGCCTTGGGGTTA

## Data Availability

The datasets generated and/or analyzed during the current study are available from the corresponding author upon reasonable request.
